# Management of Metastatic Pancreatic Cancer—Comparison of Global Guidelines over the Last 5 Years

**DOI:** 10.3390/cancers15174400

**Published:** 2023-09-02

**Authors:** Monika Pajewska, Olga Partyka, Aleksandra Czerw, Andrzej Deptała, Elżbieta Cipora, Izabela Gąska, Marek Wojtaszek, Katarzyna Sygit, Marian Sygit, Edyta Krzych-Fałta, Daria Schneider-Matyka, Anna M. Cybulska, Elżbieta Grochans, Alicja Asendrych-Woźniak, Agnieszka Romanowicz, Jarosław Drobnik, Ewa Bandurska, Weronika Ciećko, Barbara Maciuszek-Bartkowska, Mateusz Curyło, Kacper Wróbel, Remigiusz Kozłowski, Michał Marczak

**Affiliations:** 1 Department of Health Economics and Medical Law, Medical University of Warsaw, 01-445 Warsaw, Poland; mpajewska@pzh.gov.pl (M.P.);; 2 Department of Economic and System Analyses, National Institute of Public Health NIH-National Research Institute, 00-791 Warsaw, Poland; 3 Department of Oncology Propaedeutics, Medical University of Warsaw, 01-445 Warsaw, Poland; 4 Medical Institute, Jan Grodek State University in Sanok, 38-500 Sanok, Poland; 5 Faculty of Health Sciences, Calisia University, 62-800 Kalisz, Poland; 6 Department of Basic of Nursing, Faculty of Health Sciences, Medical University of Warsaw, 01-445 Warsaw, Poland; 7 Department of Nursing, Faculty of Health Sciences, Pomeranian Medical University in Szczecin, 71-210 Szczecin, Poland; 8 Clinical Department of Oncology, The National Institute of Medicine of the Ministry of Interior and Administration, 02-507 Warsaw, Poland; 9 Department of Family Medicine, Faculty of Medicine, Wroclaw Medical University, 51-141 Wroclaw, Poland; 10 Center for Competence Development, Integrated Care and e-Health, Medical University of Gdansk, 80-204 Gdansk, Poland; 11 Department of Biophysics, Poznan University of Medical Sciencies, 60-780 Poznan, Poland; 12 Department of Internal Medicine, Rehabilitation and Physical Medicine, Medical University of Lodz, 90-647 Lodz, Poland; 13 Medical Rehabilitation Department, The Ministry of the Interior and Administration Hospital, 30-053 Cracow, Poland; 14 Department of Management and Logistics in Healthcare, Medical University of Lodz, 90-131 Lodz, Poland; 15 Center for Security Technologies in Logistics, Faculty of Management, University of Lodz, 90-237 Lodz, Poland; 16 Collegium of Management, WSB Merito University in Warsaw, 03-204 Warszawa, Poland

**Keywords:** pancreatic cancer, metastases, pancreatic ductal adenocarcinoma, guidelines

## Abstract

**Simple Summary:**

Pancreatic cancer (PC) is a disease with a poor prognosis due to several factors, such as late diagnosis at an advanced stage of the disease, metastases and resistance to most treatment regimens. In recent years, only a few new treatments have been developed, but not all of them are eligible to treat all of the patients. The main objective of this review is to present the advancements in treatment guidelines from recent years.

**Abstract:**

Pancreatic cancer (PC) is usually diagnosed at an advanced stage of its development, which results in lower overall survival (OS). Prognosis is also poor even with curative-intent surgery. Approximately 80% of patients with localized PDAC have micrometastases at the time of diagnosis, which leads to a worse prognosis than in other cancers. The objective of this study is to present the progress in the treatment of metastatic pancreatic cancer based on the recommendations of oncological scientific societies, such as ESMO, NCCN, ASCO, NICE and SEOM, over the last 5 years. Combined FOLFIRINOX therapy is mostly a recommended therapy among patients with good performance statuses, while gemcitabine is recommended for more fragile patients as a first-line treatment. The newest guidelines suggest that molecular profiling of the tumor should be the first step in determining the course of treatment. The use of modern molecular therapies in patients with specific gene mutations should extend the survival of patients with this disease.

## 1. Introduction

Pancreatic cancer (PC) is usually diagnosed at an advanced stage of its development, which results in lower overall survival (OS) and progression-free survival (PFS) rates compared to other common cancers in Europe. Pancreatic cancer deaths have doubled over the last decades, and now around 95,000 deaths from PC are recorded in the EU [1]. Forecasts predict that the number of deaths may increase by 40% by 2035. In 2020, the standardized mortality rate (ESP—2013 per 100,000 population) for the EU was 27: Germany 21, France 19.6, Hungary 23.9, Austria 21.4, Poland 16.1 [2]. In Poland, in 2020, 2431 deaths were recorded due to pancreatic cancer among men, which accounted for 4.5% of deaths due to other cancers, and 2542 deaths among women, which accounted for 5.6% of deaths due to other cancers [3].

The most common histological type of pancreatic cancer is pancreatic ductal adenocarcinoma (PDAC) and, therefore, both terms PC and PDAC will be used interchangeably in this article. Pancreatic ductal adenocarcinoma is characterized by an aggressive course, deeply infiltrating the surrounding tissues and quickly resulting in distant metastases, thus causing high mortality of patients [4]. The occult clinical course and non-specific symptoms are the reason for the late diagnosis of PDAC, which usually precludes radical resection and recovery [5]. Studies indicate that approximately 80% of patients with localized PDAC have micrometastases at the time of diagnosis, as evidenced by the high rate of cancer recurrence even in patients receiving cancer treatment [6,7]. In addition, the specific tumor microenvironment, i.e., desmoplastic and strongly immunosuppressive, creates optimal conditions for the rapid development of cancer cell clones, which relatively easily become resistant to the applied systemic treatment and radiotherapy. Additionally, pancreatic cancer is highly heterogeneous at the molecular and cellular levels, and it is thus treatment-resistant [8]. It is estimated that in highly developed countries, the 5-year survival rate of patients with PDAC, from the moment of diagnosis, ranges between 8 and 14% [9,10].

The main risk factors for the development of PC include hereditary and a family history of pancreatic cancer (familial pancreatic cancer—FPC), chronic pancreatitis, the presence of intraductal papillary mucinous neoplasm (IPMN), gallstones and pancreatic cysts [11]. Familial pancreatic cancer is defined as occurring in at least two first-degree relatives with pancreatic cancer [12]. The risk of pancreatic cancer increases exponentially with the number of first-degree relatives, from three-fold when two first-degree relatives have pancreatic cancer to a 57-fold increased risk when three first-degree relatives have the disease [13].

The vast majority of PC is sporadic cancer, where lifestyle plays an important role in the development of the disease [14,15,16]. Factors such as smoking, excessive alcohol consumption, obesity and physical inactivity, as well as exposure to chemicals (e.g., benzene, petrochemicals, dyes and pesticides), are known risk factors for developing pancreatic cancer [14,16]. For example, smoking is responsible for more than 20% of all cases of pancreatic cancer [17]. Environmental tobacco smoke (ETS), also referred to as second-hand smoke, and exposure to second-hand smoke have been associated with the onset of PC [18]. ETS exposure in non-smokers before the age of 21 and in those aged 21–40 is associated with a much earlier age of onset of PC compared to non-exposed individuals; therefore, a dose–effect relationship is likely [19]. Alcohol consumption, in turn, contributes to episodes of acute pancreatitis and, subsequently, to the development of chronic pancreatitis, which is a risk factor for PC [19].

It is estimated that approximately up to 20% of all PDAC cases are hereditary [20]. Genetic factors that constitute risk factors for PC include mutations of BRCA1 or BRCA2, PALB2 and p16/CDKN2A, which is also associated with melanoma of the skin and eyes. Furthermore, familial pancreatitis is associated with a mutation in the PRSS1 gene, hereditary nonpolyposis-related colorectal cancer (HNPCC/Lynch syndrome) most often caused by a defect in the MLH1 or MSH2 gene, Peutz-Jeghers syndrome (PJS) is caused by defects in the STK11 gene and FAP syndrome is caused by a germline mutation in the APC gene [21,22,23,24,25]. Some studies also indicate a possible association between Helicobacter pylori or HBV infection and the development of PC [26,27,28]. Thanks to advances in sequencing technology, it is possible to study the diversity of mutations leading to the development of the disease. However, approximately 80–90% of the genetic events leading to familial pancreatic cancer remain unknown, and only 10–20% have a uniquely identifiable germline mutation. This makes it difficult to properly distinguish FPC from apparently sporadic ductal adenocarcinoma. Exome and DNA copy-number variations (CNV) analyses in PDAC revealed a complex mutational background in this disease. KRAS gene-activating mutations are very common (over 90%) and inactivation of TP53, SMAD4 and CDKN2A occurs in over 50% of cases [29,30]. The incidence of recurrent gene mutations then drops to ~10% for genes involved in chromatin remodeling, DNA damage repair and other mechanisms known to play an important role in the process of carcinogenesis [29].

The heterogeneity of molecular disorders found in pancreatic cancer and the desmoplastic and immunosuppressive effects of the tumor microenvironment mean that the possibilities of effective treatment of patients with metastatic PDAC are limited. So far, classical chemotherapy has proved to be the basic method that allows for improving the survival of patients with metastatic pancreatic cancer (mPC) and metastatic pancreatic ductal adenocarcinoma (mPDAC) [31]. In molecularly defined subtypes of PC, survival can be further extended with appropriate targeted therapies; however, their value needs to be confirmed in prospective randomized phase III studies with a random selection of patients. The identification of genetic biomarkers in mPDAC is essential for the further development of targeted therapies. The objective of this study is to present the progress in the treatment of metastatic pancreatic cancer that has been made over the last 5 years, based on the recommendations of the most important oncological scientific societies.

## 2. Materials and Methods

Based on the analysis of the guidelines of leading international organizations and scientific societies, and the results of randomized clinical trials, a summary of the state of knowledge and progress in the treatment of metastatic PDAC was prepared, taking into account the recommendations of various scientific societies. In order to present changes over the last 5 years, the recommendations of the American Society of Clinical Oncology (ASCO), the European Society for Medical Oncology (ESMO), the Spanish Society of Medical Oncology (Sociedad Española de Oncología Médica—SEOM) and the National Institute for Health and Care Excellence (NICE, UK) were used. As the above recommendations have not been updated for several years, they were compared with the latest National Comprehensive Cancer Network (NCCN, USA) study from 2023.

## 3. Results

The table below presents a summary of the guidelines from the last 5 years.

### 3.1. Advances in mPDAC Therapy over the Years

For many years, gemcitabine (GEM), introduced in 1996, was the standard systemic therapy available to patients with mPDAC [31]. The median survival for GEM monotherapy was 5.65 months compared to 4.41 months for the then used 5-fluorouracil (5-FU) [32]. Despite a slight prolongation of overall survival, gemcitabine has become a standard in the treatment of mPDAC, as it significantly improved the clinical benefit rate. Soon, research on drugs was commenced to improve the results obtained in monotherapy. In 2007, in a study by Moore et al., GEM was compared with the combination of gemcitabine and erlotinib (a potent and selective epidermal growth factor receptor (EGFR) tyrosine kinase inhibitor) [33]. Scientific evidence has suggested that the overexpression of EGFR is associated with a poor prognosis of pancreatic cancer. The results of this study showed a statistically significant, but not clinically significant, improvement in survival of 0.33 months in favor of the combination therapy. However, patients who developed CTC-NCI grade > G1 skin rash lived longer: median OS was 11 months [33].

In 2011, Conroy et al. published a randomized and prospective phase III study that compared the use of FOLFIRINOX (5-FU infused over 46 h, biomodulated with folinic acid, plus irinotecan and oxaliplatin) with gemcitabine monotherapy in patients with mPDAC [34]. A statistically significant prolongation of survival was demonstrated in the group of patients aged 25–76 (median 61) who received FOLFIRINOX: up to 11.1 months versus 6.8 months in GEM monotherapy. This study was groundbreaking, because for the first time a significant prolongation of survival and high rates of objective responses were obtained; however, it should be emphasized that the greatest benefits from prolonging the OS concerned patients aged ≤ 76 and with good performance status (ECOG 0-1).

In 2013, Von Hoff et al. published the results of another randomized and prospective phase III study in mPDAC where patients with good performance status (Karnofsky ≥ 70%), aged 27–88 (median 63), were randomized to receive nab-paclitaxel with gemcitabine (nab-PXL-GEM) or gemcitabine monotherapy [35]. The median overall survival was 8.5 months in combination therapy versus 6.7 months in GEM monotherapy [3].

Indirectly comparing these two groundbreaking studies, it should be emphasized that the FOLFIRINOX regimen showed longer OS and PFS and a higher rate of clinical responses but also a higher frequency of hematological adverse events and diarrhea than nab-PXL-GEM therapy. In contrast, the nab-PXL-GEM regimen resulted in a higher rate of sensory and motor polyneuropathy compared to the FOLFIRINOX regimen. According to the quoted guidelines (Table 1), treatment with the FOLFIRINOX regimen is recommended primarily for patients with good performance status (according to ECOG = 0–1) and below the age of 76 years, and for patients with an ECOG performance status = 2 or with comorbidities, gemcitabine monotherapy remains the recommended treatment. The nab-PXL-GEM regimen is generally used in patients who have contraindications to receive the FOLFIRINOX regimen. Thus, when choosing first-line chemotherapy, not only the extent of mPDAC should be taken into account but also the general condition of the patient and the presence of comorbidities.

**Figure 1:**
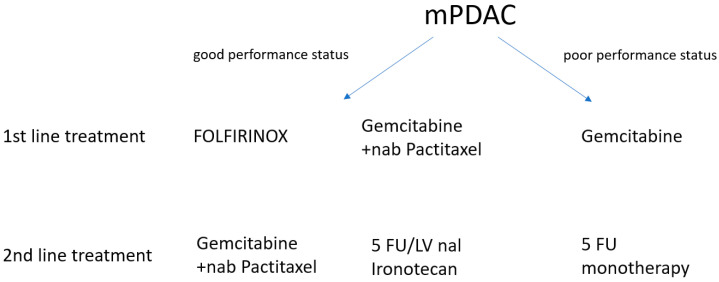
Classical chemotherapy in the treatment of metastatic pancreatic cancer.

In the international randomized phase III study NAPOLI-1, patients after the progression of gemcitabine-based chemotherapy received monotherapy with nal-IRI (nanoliposomal irinotecan) at a dose of 120 mg/m^2^ every 3 weeks or 5-fluorouracil (2000 mg/m^2^) and leucovorin (200 mg/m^2^) weekly for the first 4 weeks of therapy in 6-week cycles and nal-IRI (800 mg/m^2^) every two weeks with 5-FU (2400 mg/m^2^) and LV (400 mg/m^2^) also every two weeks [36]. The results of the study showed higher OS for nal-IRI + 5-FU/LV therapy (6.2 months) compared to 5-FU/LV (4.2 months), HR = 0.75. No significant differences in OS were observed with nal-IRI monotherapy (4.9 months) and 5-FU/LV (4.2 months). The Kaplan-Meier OS curves converged close to the 20-month point; survival exceeded this result for less than 10% of patients. For nal-IRI + 5-FU/LV patients, the median PFS was 3.1 months versus 1.5 months for 5-FU/LV patients. In contrast, for nal-IRI monotherapy, PFS was 2.7 months compared to 1.6 months for the 5-FU/LV regimen [36]. Standard recommended chemotherapy treatment is presented in the figure below (Figure 1).

Treatment options depend on the molecular profile of the tumor. In 2008, Jones et al. published the results of the study, where a comprehensive genetic analysis of 24 pancreatic cancers was performed [37]. The researchers determined 69 gene sets that were genetically altered in the majority of the examined PC cases, defining a core set of 12 cellular signaling pathways. Those results provided the data required for personalized cancer medicine. In 2011, Collisson et al. conducted a combined analysis of transcriptional profiles of primary PDAC samples from several studies along with human and mouse PDAC cell lines [38]. As a result, subtypes and gene signatures for those subtypes of PC were identified. Another significant study aimed at an investigation of primary and metastatic PDAC gene expression using NMF (NMF–nonnegative matrix factorization) and was conducted in 2015 by Moffitt et al. [39]. Those studies provided new insight into the molecular composition of PDAC and laid the foundations for the development of modern targeted therapies. However, the first success of targeted therapy was noted only in 2019, when the results of a randomized, double-blind study with the acronym POLO (Pancreas Cancer Olaparib Ongoing) were published [40]. The objective of this study was to evaluate the efficacy of maintenance therapy with olaparib (a PARP inhibitor) in patients with germline BRCA 1 and 2 mutations and metastatic pancreatic adenocarcinoma without progression during first-line platinum-based chemotherapy. The results showed that olaparib had a longer PFS (7.4 months) compared to placebo after platinum-based chemotherapy (3.8 months), but there was no significant difference in OS (median 18.9 months versus 18.1 months, respectively) [40]. The results of the study were subjected to a broad scientific discussion, in which many experts emphasized that the lack of improvement in OS could be due to the use of subsequent therapies (including PARP inhibitors) in the placebo group, which were administered to patients with mPDAC after progression.

### 3.2. Changes in Guidelines over the Years

In the first-line treatment, the FOLIFRINOX regimen is recommended by ESMO 2019, ASCO 2018–2020, NICE 2018, SEMO 2020 as well as NCCN 2023 [35,36,37,38]. It should be noted, however, that due to the increased toxicity profile (especially hematological—higher risk of febrile neutropenia) in this treatment regimen, it is appropriate for patients in better overall health (ECOG 0-1) as highlighted in the guidelines above (Table 1).

Gemcitabine monotherapy as a first-line treatment is recommended by ESMO 2019, ASCO 2018, ASCO 2020, SEMO 2020 and NCCN 2023 for patients with poorer performance status (ECOG 2) and with systemic comorbidities (ischemic heart disease, COPD, insufficient control of hypertension, chronic kidney disease grades G1-G2, mild liver failure defined by bilirubin < 3 mg/dL and liver enzymes < 5× ULN, etc.) in whom the risk of complications resulting from FOLFIRINOX chemotherapy exceeds the expected benefits in the form of prolonged OS, because they are unable to tolerate the toxicity of a multidrug regimen. Gemcitabine in combination with capecitabine appeared as a recommendation in the ESMO 2019 guidelines for patients with mPDAC who do not have any contraindications to the use of 5-fluoropyrimidines but do have contraindications to oxaliplatin and irinotecan, and therefore are not eligible for treatment with the FOLFIRINOX regimen. It should be noted, however, that the effect of the gemcitabine/capecitabine regimen on improving OS is illusory.

In turn, in the NCCN 2023 guidelines, there is a recommendation to perform genetic/molecular tests, when determining the first-line systemic treatment, which is to help qualify patients for clinical trials at this stage of therapy or to facilitate the selection of a therapeutic strategy based on molecular profiling after progression during the first-line treatment. This is a significant change compared to the guidelines of other societies from previous years because it takes into account the scientific achievements resulting from the publication of the results of randomized and prospective clinical trials after 2020. In 2020, ASCO, for the first time, recommended early testing of applicable genomic alterations in guidelines for patients who would be potential candidates for molecularly targeted therapies after first-line chemotherapy. According to the recommendations, molecular testing should be aimed at detecting BRCA gene mutations, microsatellite instability and NTRK gene fusions. Genetic testing should be performed as part of the initial assessment so that results are available in a timely manner for subsequent clinical decisions. In 2020, the use of entrectinib was included in the ASCO guidelines for the first time. According to studies, this drug induced durable and clinically significant responses in NTRK fusion-positive solid tumor patients [39]. Pembrolizumab (an anti-PD-1 monoclonal antibody) and olaparib (a PARP inhibitor) were included in the ASCO guidelines for the first time in 2020 for patients with mPDAC, with microsatellite instability and BRCA mutations, respectively. This is the American society’s response to the lack of a sufficiently effective treatment. The inclusion of these innovative therapies in the guidelines allows for the extension of patients’ survival.

**Figure 2:**
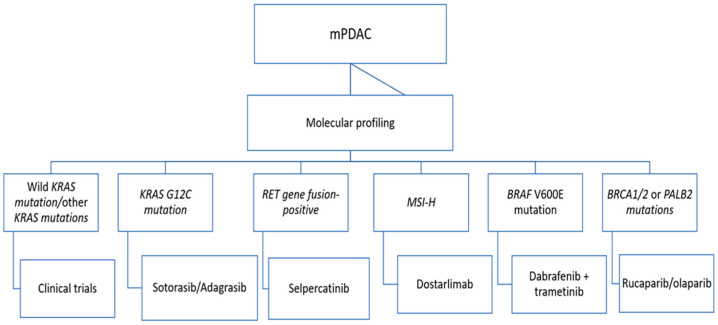
Targeted treatment strategy in metastatic pancreatic cancer.

In the NCCN 2023 recommendations, a group of patients whose mPDAC shows characteristic KRAS gene mutations (KRAS G12C) have a chance to extend survival as a result of using molecular therapies with sotorasib or adagrasib. In addition to pembrolizumab, for the first time, another anti-PD-1 monoclonal antibody appeared in these recommendations for the treatment of mPDAC patients with microsatellite instability (see Figure 2).

Gemcitabine in combination with nab-paclitaxel is recommended as the second-line treatment in the ASCO 2018, SEMO 2020, NICE 2018 and ESMO 2019 guidelines—i.e., in patients previously treated with the FOLFIRINOX regimen—while the 5-FU + nal-Iri regimen is reserved for patients with mPDAC progression after gemcitabine.

### 3.3. Future Research and Use of Bioinformatics

Due to the complicated course of pancreatic cancer, it is important to search for new methods of early diagnosis in order to improve OS and quality of life of patients. It is worth mentioning that properly prepared bioinformatics tools can significantly support the process of identifying new biomarkers and the prognosis of the course of the disease. In a study by Vandenbrouck et al., specifically designed bioinformatic tools were used to identify 24 potential biomarkers for early PDAC detection [41]. The tools used in this study were part of proteomics research environment (ProteoRE), a Galaxy-based instance dedicated to the functional analysis and exploration of proteomics data in biomedical research. In the study by Shi et al., a bioinformatic approach was used in order to identify the role of the associated genes in the development and progression of PDAC and determine relevant molecular markers for early detection and targeted therapies [42]. Another study by Ke et al. conducted biological behavior experiments using bioinformatics tools [43]. Based on the analyses, the authors concluded that DLGAP5 is most closely associated with survival in PC and may be used as a prognostic indicator. However, it is worth noting that the biological role of gene expression in pancreatic cancer still requires further study. A study conducted by Xu et al. focused on the molecular mechanisms responsible for metastasis in pancreatic cancer [44]. Researchers pointed to a possible important role of the IGFBP1 gene in the process of metastasis. Furthermore, this is important information that allows for further developing research on the mechanism of metastasis in pancreatic cancer.

## 4. Conclusions

Recommendations of international oncological societies from previous years (ASCO, ESMO, NICE, SEOM) confirm progress, albeit small, in the treatment of patients with metastatic pancreatic cancer [45,46,47,48] Comparing these recommendations with the latest ones (NCCN 2023), it can be concluded that there are no significant differences in the field of classic chemotherapy. The hope for longer survival of mPDAC patients is in the development of targeted molecular therapies, which have begun to appear in ASCO 2020 recommendations and in a wider range in NCCN 2023 recommendations. Further research is undoubtedly needed, especially concerning phase III (prospective and randomized with a random selection of patients), using classical chemotherapy, immunotherapy and molecularly targeted drugs, the results of which would translate into the longer survival of patients with metastatic pancreatic cancer. Beyond molecular profiling, functional profiling using patient-derived PDAC organoids might be helpful in predicting the resistance vs. responsiveness of individual PDAC to chemotherapeutic and personalized medicine-based therapy options [49].

## Figures and Tables

**Table 1:** Comparison of mPDAC treatment guidelines.

ESMO 2019	ASCO 2018	ASCO 2020	SEOM 2020	NICE 2018	NCCN 2023
First-line treatment mFOLFIRINOX as the first option of adjuvant treatment after the resection of pancreatic cancer in selected (good performance) patients due to the survival results and the related toxicity profile; In more weakened patients (age > 70 years, ECOG performance status or patients with contraindications to the drugs used in FOLFIRINOX), gemcitabine/capecitabine; Gemcitabine monotherapy in patients with poorer overall health; Second-line treatment Nanoliposomal irinotecan with fluorouracil and folinic acid is recommended for metastatic patients previously treated with gemcitabine-based therapy;	First-line treatment FOLFIRINOX in patients who meet the following criteria: ECOG 0-1, favorable comorbidity profile, a support system for aggressive medical therapy and access to a chemotherapy port and infusion pump management service; Gemcitabine plus nab-paclitaxel for patients who meet the following criteria: ECOG 0-1, a relatively favorable comorbidity profile; Gemcitabine monotherapy is recommended for patients with an ECOG PS of 2 or a comorbidity profile that precludes more aggressive regimens and who wish to continue cancer-targeted therapy. Adding capecitabine or erlotinib to gemcitabine may be proposed; Patients with an ECOG PS ≥ 3 or with poorly controlled comorbidities despite continued active medical care should only receive targeted therapy on a case-by-case basis. The main focus should be on optimizing supportive care; Second-line treatment Gemcitabine with nab-paclitaxel for patients who meet all of the following criteria: first-line treatment with FOLFIRINOX, ECOG PS 0 to 1, relatively favorable comorbidity profile, and patient preference and support for aggressive medical therapy; Fluorouracil with nanoliposomal irinotecan or fluorouracil in combination with irinotecan in patients who meet the following criteria: first-line gemcitabine plus nab-paclitaxel, ECOG PS 0 to 1, a relatively favorable comorbidity profile, patient preferences and a support system for aggressive medical therapy and access to a chemotherapy port and infusion pump management services; Fluorouracil with oxaliplatin in patients who meet the following criteria: first-line treatment with gemcitabine in combination with nab-paclitaxel, ECOG PS 0 to 1, relatively favorable comorbidity profile, patient preference and support system for aggressive medical therapy, and access to a chemotherapy port and infusion pump management services; Gemcitabine or fluorouracil for patients with an ECOG PS of 2 or a comorbidity profile that precludes more aggressive regimens and who wish to continue therapy.	First-line treatment In patients who will be potential candidates for additional treatment after first-line therapy, early screening for applicable genomic changes is recommended. Both germline and tumor testing are recommended. Other guidelines as in ASCO 2018; Second-line treatment Treatment with larotrectinib or entrectinib is recommended in patients with NTRK fusions; Programmed immune checkpoint-death-1 inhibitor—pembrolizumab is recommended as second-line therapy for patients who test high for mismatch repair deficiency or microsatellite instability; For patients with a BRCA1 or BRCA2 mutation who have received first-line platinum-based chemotherapy without disease progression for at least 16 weeks, further treatment options include chemotherapy or the PARP inhibitor—olaparib.	First-line treatment FOLFIRINOX/mFOLFIRINOX and/or nab-paclitaxel in patients with ECOG/PS 0-1 and below the age of 75; Gemcitabine and gemcitabine in combination with nab-paclitaxel in selected patients with an ECOG score of 2 or > 75 years should be considered; Combination of nal-IRI and 5-FU alternative for patients transitioning to gemcitabine-based chemotherapy, where available; Second-line treatment Gemcitabine and nab-paclitaxel in fit patients after 5-FU-based chemotherapy; In patients with a BRCA1/BRCA2 mutation, first-line platinum-based chemotherapy followed by olaparib maintenance therapy;	First-line treatment FOLFIRINOX for patients with ECOG 0–1; Combination therapy with gemcitabine in patients who cannot tolerate FOLFIRINOX; Gemcitabine monotherapy for patients who are not healthy enough to tolerate combination chemotherapy; Second-line treatment Oxaliplatin-based chemotherapy as second-line treatment for people who have not received first-line oxaliplatin; Gemcitabine-based chemotherapy as second-line treatment for people whose cancer is progressing after first-line treatment with FOLFIRINOX; Nab-paclitaxel (Albumin-bound nanoparticle paclitaxel) with gemcitabine recommended for untreated adult metastatic pancreatic adenocarcinoma provided that Other combination chemotherapies are unsuitable and gemcitabine monotherapy would otherwise be used.	First-line treatment If jaundice is present: the application of SEMS (self-expandable metal stent); Genetic testing for hereditary mutations if not previously tested; Molecular profiling of the tumor tissue, if not performed, if the patient’s condition is good or moderately good—qualification for a clinical trial (preferred) or systemic therapy (FOLFIRINOX or modified FOLFIRINOX followed by chemoradiotherapy or gemcitabine + albumin-bound paclitaxel ± followed by radiochemotherapy) if BRCA1/2 or PALB2 mutation is present—FOLFIRINOX or modified FOLFIRINOX, followed by chemoradiotherapy or Gemcitabine + cisplatinum (≥2–6 cycles) ± then chemoradiotherapy, if there is no progression of the disease after 4–6 months (at an acceptable level of toxicity), we continue the therapy from the clinical trial; in the case of poor general condition of the patient, palliative therapy or monochemotherapy or targeted therapy based on molecular profiling, as clinically indicated, or palliative radiotherapy; Maintenance therapy Use FOLFIRINOX or modified FOLFIRINOX followed by chemoradiotherapy or gemcitabine + albumin-bound paclitaxel ± then radiochemotherapy or if there is a BRCA1/2 or PALB2 mutation—FOLFIRINOX or modified FOLFIRINOX followed by chemoradiotherapy or Gemcitabine + cisplatin (≥2–6 cycles) ± then chemoradiotherapy or stop chemotherapy, if the disease progresses, the progression scheme is used if the previous therapy was based on platinum: Rucaparib (for germline or somatic BRCA1/2 or PALB2 mutations); Further therapy of metastatic disease: ntrectinib (if NTRK gene fusion is positive) Larotrectinib (if NTRK gene fusion is positive) Pembrolizumab (if MSI-H, dMMR or TMB-H [≥10 mut/Mb]) Dabrafenib + trametinib (if BRAF V600E mutation positive) Dostarlimab (if MSI-H or dMMR) Selpercatinib (if RET gene fusion is positive) Sotorazib/Adagrasib (for KRAS G12C mutation) Nivolumab + ipilimumab (if TMB-H [≥10 mut/Mb]); If previous therapy was based on gemcitabine, the following is recommended: 5-FU + leucovorin + liposomal irinotecan Capecitabine CapeOx 5-FU continuous infusion FOLFIRI FOLFIRINOX or modified FOLFIRINOX FOLFOKS OFF (oxaliplatin, folinic acid and 5-fluorouracil)

## Data Availability

No new data were created during the preparation of this manuscript.
